# Highly purified and functionally stable *in vitro* expanded allospecific Tr1 cells expressing immunosuppressive graft-homing receptors as new candidates for cell therapy in solid organ transplantation

**DOI:** 10.3389/fimmu.2023.1062456

**Published:** 2023-02-24

**Authors:** Saúl Arteaga-Cruz, Arimelek Cortés-Hernández, Evelyn Katy Alvarez-Salazar, Katya Rosas-Cortina, Christian Aguilera-Sandoval, Luis E. Morales-Buenrostro, Josefina M. Alberú-Gómez, Gloria Soldevila

**Affiliations:** ^1^ Department of Immunology, Biomedical Research Institute, Mexico City, Mexico; ^2^ The National Laboratory of Flow Cytometry, Biomedical Research Institute, National Autonomous University of Mexico, Mexico City, Mexico; ^3^ Scientific affairs, Accellix, San Jose, CA, United States; ^4^ Department of Nephrology and Mineral Metabolism, National Institute of Medical Sciences and Nutrition Salvador Zubirán, Mexico City, Mexico; ^5^ School of Medicine and Health Sciences, Tecnológico de Monterrey., Mexico City, Mexico

**Keywords:** Type 1 Tregs, allospecific, expansion, transplantation, tolerance, inflammatory cytokines

## Abstract

The development of new strategies based on the use of Tr1 cells has taken relevance to induce long-term tolerance, especially in the context of allogeneic stem cell transplantation. Although Tr1 cells are currently identified by the co-expression of CD49b and LAG-3 and high production of interleukin 10 (IL-10), recent studies have shown the need for a more exhaustive characterization, including co-inhibitory and chemokines receptors expression, to ensure bona fide Tr1 cells to be used as cell therapy in solid organ transplantation. Moreover, the proinflammatory environment induced by the allograft could affect the suppressive function of Treg cells, therefore stability of Tr1 cells needs to be further investigated. Here, we establish a new protocol that allows long-term *in vitro* expansion of highly purified expanded allospecific Tr1 (^Exp-allo^ Tr1). Our expanded Tr1 cell population becomes highly enriched in IL-10 producers (> 90%) and maintains high expression of CD49b and LAG-3, as well as the co-inhibitory receptors PD-1, CTLA-4, TIM-3, TIGIT and CD39. Most importantly, high dimensional analysis of ^Exp-allo^ Tr1 demonstrated a specific expression profile that distinguishes them from activated conventional T cells (T conv), showing overexpression of IL-10, CD39, CTLA-4 and LAG-3. On the other hand, ^Exp-allo^ Tr1 expressed a chemokine receptor profile relevant for allograft homing and tolerance induction including CCR2, CCR4, CCR5 and CXCR3, but lower levels of CCR7. Interestingly, ^Exp-allo^ Tr1 efficiently suppressed allospecific but not third-party T cell responses even after being expanded in the presence of proinflammatory cytokines for two extra weeks, supporting their functional stability. In summary, we demonstrate for the first time that highly purified allospecific Tr1 (^Allo^ Tr1) cells can be efficiently expanded maintaining a stable phenotype and suppressive function with homing potential to the allograft, so they may be considered as promising therapeutic tools for solid organ transplantation.

## 1 Introduction

T regulatory Type one (Tr1) cells are a subset of CD4^+^ T cells initially described in patients with mixed chimerism who did not develop Graft vs Host Disease (GvHD), after hematopoietic stem cell transplantation (HSCT) (1, 2). Several reports have shown that Tr1 cells can be abundantly found in specific anatomical regions with IL-10-rich microenvironments such as the intestinal mucosa (3), promoting the tolerance induction towards microbiota and diet-derived antigens (4). Nonetheless, the presence of Tr1 cells can be identified in peripheral blood and spleen, indicating that they can exert their functions both locally and systemically (5).

Tr1 cells are characterized by the co-expression of the specific surface markers: CD49b and LAG-3 (lymphocyte activation gene-3) which has facilitated their identification and purification (6). Another relevant aspect of these cells is their capacity to produce high and intermediate amounts of IL-10 and TGF-β respectively, variable levels of IFN-γ and IL-2 and low or null production of IL-4 (reviewed in (7)). Although the production of anti-inflammatory cytokines has been described as one of their main suppressive mechanisms, the ability to exert cell-cell contact suppression (PD1 and CTLA-4), metabolic disruption (CD39 and CD73), as well as cytotoxicity (Granzyme B and perforin release) are other important features which regulate effector immune responses (reviewed in (8)). Importantly, Brockmann and co-workers have recently described a subpopulation within the CD49b^+^LAG-3^+^CD4^+^ Tr1 cells which co-expresses the co-inhibitory receptors (CIR) TIGIT, TIM-3 and PD1, and produce higher levels of IL-10. Moreover, CCR5+ CIR^+^ Tr1 cells appear to have greater immunoregulatory capacity (9).

Tr1 cells had previously been used as therapy tools in different preclinical models of intestinal inflammation (10) and autoimmune diseases, where they contribute to resolve effector T cell responses and reduce the clinical score disease (11–13). However, most of their therapeutic potential has been described within the allograft context, where *in vitro* differentiated Tr1 cells were able to efficiently induce tolerance towards allogeneic pancreatic islets and prevented GvHD development in HSCT patients (reviewed in (14)). In this context, IL-10-anergized donor T cells (IL-10-DLI) were able to promote cellular reconstitution in HSCT transplanted patients while reducing the risk of GvHD (15). Other protocols based on T10-cells, a clinical grade population obtained after stimulation of CD4^+^ T cells with irradiated IL-10 derived allogeneic dendritic cells (DC_10_), have been safely used in patients with kidney transplant. Importantly, circulating T10-cells showed a stable tolerogenic gene signature thirty-six weeks post-transplantation (16). Recently, a new protocol focused on HSC transplant patients with hematological malignancies used a population obtained from CD4^+^ T cells cultured with allogeneic DC_10_ in presence of IL-10 (T-allo10) as cell therapy, showing the maintenance of a stable population after patient’s infusion (17).

One of the important aspects to consider for the use of cellular therapy in transplanted patients is to ensure the stability of infused regulatory T (Tregs) cells in the transplanted patient. In this context, it has been reported that activation of thymic and induced Tregs cells expressing FOXP3 in the presence of proinflammatory cytokines, can promote a downregulation of FOXP3, leading to a loss of their suppressive capacity, while acquiring characteristics from inflammatory T cell populations ( (18) and reviewed in (19)). On the other hand, there is growing evidence that chemokine receptor expression in infiltrated allograft cells is closely related to either the rejection or maintenance of organ transplants, as previously reported for FOXP3^+^ Tregs cells (20). However, to date, scarce information is available about the effect of inflammatory cytokines and chemokine receptor expression on Tr1 stability and homing, respectively. In addition, it has been demonstrated that specific chemokine receptors (CR) expressed on Tregs cells, play a key role in transplantation tolerance, by favoring their homing Tregs cells towards the allograft (CCR2, CXCR3 and CCR4) or draining lymph nodes (CCR7) (20, 21). Initial studies reported the expression of CR related to both Type 1 helper (Th1) (CXCR3 and CCR5) and (Th2) subsets (CCR3, CCR4 and CCR8), while CCR5 has been proposed as a phenotypic marker for Tr1 cells (9). However, a more detailed characterization is still needed to evaluate their functional relevance in allograft tolerance.

Although current protocols to differentiate alloantigen-specific Tr1 cells *in vitro* have shown satisfactory results (15–17), none of these protocols have successfully obtained pure allospecific Tr1 cells and, furthermore, new methodologies are needed aiming at large-scale Tr1 cell production. In the present work, we describe for the first time the feasibility of large scale allospecific Tr1 (^Allo^ Tr1) cells expansion, with high purity and stable function as potential tools for solid organ transplantation.

## 2 Materials and methods

### 2.1 Healthy donor samples

Buffy coats products from healthy donors whole blood were collected in the Blood Bank of “Instituto Nacional de Enfermedades Respiratorias (INER) Ismael Cosío Villegas”, Mexico City, after written informed consent, in accordance with the local human ethics committee procedures. This protocol was approved by the Committees Medical Ethics at the Instituto de Investigaciones Biomédicas (UNAM) and the Instituto Nacional de Ciencias Médicas y Nutrición Salvador Zubirán (Reference #1831) and performed in accordance with the revised Declaration of Helsinki, the Declaration of Istanbul and Good Clinical Practice Guidelines.

### 2.2 Reagents and antibodies

For flow cytometry analysis, APC-Cy7 anti-CD4, PE-Cy7 anti-CD8, APC anti-CD11c and FITC anti-CD14 (San Diego, CA, USA), PE Cy7 anti-LAG-3, PE-Cy5.5 anti-CD3 and PerCP-eFluor 710 anti-ILT4 were purchased from Invitrogen (Waltham, MA, USA). Alexa Fluor 488 anti-PD1, Alexa Fluor 488 anti-CCR5, PerCP Cy5.5 anti-CCR7, PerCP-Cy5.5 anti-TIM3, PE anti-CD25, PE Dazzle 594 anti-TIGIT, PE-Cy7 anti-HLA-G, APC anti-CD49b, APC Fire 750 anti-CD45RA, Brilliant Violet (BV) 711 anti-CD39, BV421 anti-CTLA-4, BV421 anti-CCR4, BV711 anti-CXCR3, Zombie NIR and Zombie Aqua™, were purchased from Biolegend (San Diego, CA, USA) (**Supplementary Table 1**).

For *in vitro* experiments, Ficoll^®^ Paque Plus (Ficoll) and dimethyl sulfoxide (DMSO) were obtained from Sigma-Aldrich (San Luis, MO USA). Recombinant human (rh) GM-CSF, IFN-γ, IL-1β, IL-2, IL-4, IL-6, IL-10 and TNF-α cytokines were obtained from PeproTech (New Jersey, NY, USA). Carboxy Fluorescein Succinimidyl Ester (CFSE), CellTrace™ Violet (CTV), Dynabeads Human T-activator CD3/CD28 (anti-CD3/CD28-coated beads), DynaMag-5™ Magnet (DynaMag), CTS™ OpTmizer™ T Cell Expansion SFM medium (Expansion culture medium), RPMI 1640 medium, antibiotic-antimycotic 100X, L-glutamine (GlutaMAX™), sodium pyruvate (100 mM), MEM Non-Essential amino acids 100X and Fetal Bovine Serum (FBS) were purchased from Thermo Fisher Scientific (Waltham, MA, USA). Pooled human AB serum (AB-HS) was obtained from Gemini Bio Products (Sacramento, CA, USA). All culture mediums were supplemented with L-glutamine, sodium pyruvate, MEM-NEAA and antibiotic-antimycotic. T cell cultures were performed in round bottom 96-well culture plates (Corning, Avon, France).

### 2.3 Peripheral blood mononuclear cells isolation

Peripheral blood mononuclear cells (PBMCs) were isolated from buffy coat preparations of adult normal healthy donors by density-gradient centrifugation over Ficoll according to the manufacturer’s instructions. Isolated cells were resuspended in a solution containing 90% Fetal Bovine Serum (FBS) and 10% Dimethyl Sulfoxide (DMSO), were stored at -70°C for 24 hours and then cryopreserved to -195°C in liquid nitrogen. For functional assays, cryopreserved cells were thawed in a 37°C water bath and washed three times with culture medium supplemented with 10% FBS.

### 2.4 Monocytes-derived dendritic cells and IL-10-producing dendritic cells *in vitro* differentiation

CD14^+^ monocytes cells were isolated from PBMCs using the Human CD14 MicroBeads kit (Miltenyi Biotec, Bergisch Gladbach, Germany) according to the manufacturer’s instructions. CD14^+^ monocytes were resuspended in RPMI 1640 culture medium supplemented with 10% AB-HS, stimulated with rhIL-4 (50 ng/mL) and rhGM-CSF (50 ng/mL) in the presence (DC_10_) or absence (moDC) of rhIL-10 (10 ng/mL) and they were cultured (37°C/5% of CO_2_) for 8 days; on days 3 and 5, culture medium and cytokines (25 ng/mL rhIL-4, 25 ng/mL rhGM-CSF and 10 ng/mL rhIL-10) were refreshed. The moDC and DC_10_ phenotypes were evaluated by flow cytometry staining with Zombie Aqua (viability) and anti-CD11c, anti-CD14, anti-HLA-G and anti-ILT4 monoclonal antibodies (mAbs). Samples were acquired on a Attune NxT Cytometer (Thermo Fisher Scientific) and data were analyzed with FlowJo v10.8 software (BD Biosciences, CA, USA).

### 2.5 *In vitro* differentiation and purification of allospecific Tr1 cells

CD4^+^CD25^-^CD45RA^+^ naïve T (nT) cells were purified from PBMCs using a FACS Area I sorter (BD Biosciences, CA, USA). Sorted nT cells were labeled with CTV according to the manufacturer’s instructions and co-cultured for 14 days with moDCs (conventional T cell condition) or DC_10_ (Tr1 cell condition) at a ratio of 10:1 (nT: DC_10_ or moDC) in presence or absence of rhIL-10 (10 ng/mL), respectively; on 7 day of co-culture, a restimulation with rhIL-10 (10 ng/mL) and rhIL-2 (50 U/mL) was performed. Tr1 cell phenotype (CD4^+^CD49b^+^LAG-3^+^ and IL-10 production) was evaluated on the 14th day of co-culture by flow cytometry. IL-10 production of T cells was determined using IL-10 secretion assay and CytoStim™ kits (Miltenyi Biotec), according to the manufacturer’s instructions, with some modifications.

After 14 days of co-culture, CD4^+^CD49b^+^LAG-3^+ Allo^ Tr1 cells from nT:DC_10_ co-culture and CD4^+^ allospecific conventional T (T conv) cells from nT:moDC co-culture were purified from proliferating cells (CTV^-^) using a MoFlo XDP cell sorter (Beckman Coulter, Brea, CA, USA). Sorted ^Allo^ Tr1 cells and T conv were cultured for 2 days in Expansion culture medium (50x10^3^/well) supplemented with 10% AB-HS and IL-2 (50 U/mL) previous to the polyclonal expansion protocol.

### 2.6 Polyclonal expansion and antibody staining of Tr1 and conventional T cells

**Figure 1:**
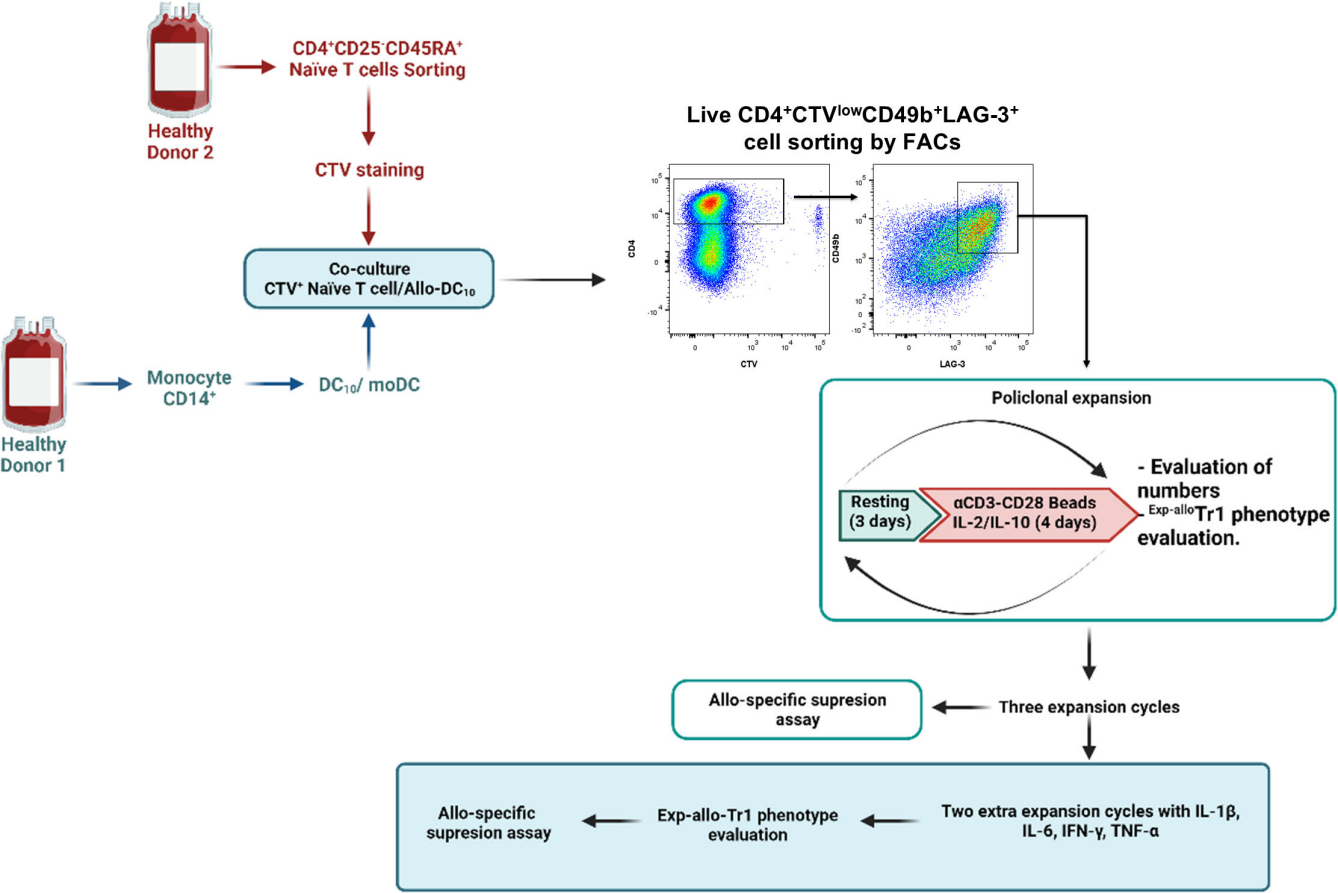
General methodological scheme used for the expansion of ^Allo^ Tr1 regulatory type 1 cells. FACS-sorted CD4^+^CD25^-^CD45RA^+^ nT cells (labeled with CTV) were stimulated with allogeneic DC_10_ in the presence of exogenous IL-2 and IL-10 for 14 days. Proliferating ^Allo^ Tr1 cells **(**CD49b^+^LAG-3^+^CTV^-^) were purified by FACs from co-cultures (CD4^+^ nT cells:DC_10_) and polyclonally-expanded for three cycles of stimulation (4 days of culture with anti-CD3/anti-CD28, rhIL-2 and rhIL-10)/resting (3 days of culture with only rhIL-2) of 7 days each. ^Exp-allo^ Tr1 cell phenotype was evaluated at each cycle of stimulation. *In vitro* functional assays were performed after three rounds of polyclonal expansion. Finally, long-term ^Exp-allo^ Tr1 cells were re-stimulated for two additional cycles in the presence of proinflammatory cytokines for Tr1 stability assays. Created by BioRender.com.

FACS-sorter Allogenic Tr1 (^Allo^ Tr1) cells (20x10^3^/well) were cultured for 4 days (37°C/5% of CO_2_) in Expansion culture medium supplemented with 10% AB-HS and stimulated with anti-CD3/CD28-coated beads at a 1:5 ratio (Beads : Tr1) plus rhIL-10 (10 ng/mL) and rhIL-2 (250 U/mL) (expansion period). After 4 days of stimulation, the anti-CD3/CD28-coated beads were removed using the DynaMag and ^Allo^ Tr1 cells (50x10^3^) were rested for 3 days in Expansion culture medium supplemented with 10% AB-HS and rhIL-2 (50 U/mL) (resting period). Three consecutive rounds of stimulation/resting (4 days of expansion and 3 days of resting each) were performed. A schematic representation of protocol is shown in **Figure 1**.

As a control group, allospecific CD4^+^ T conv cells (20x10^3^/well) were polyclonally-expanded in parallel cultures with anti-CD3/anti-CD28 coated beads at 1:5 ratio (Beads:T conv) and rhIL-2 (100 U/mL) with the same rounds of stimulation/resting used for ^Allo^ Tr1 cells.

Throughout the *ex vivo* expansion protocol, IL-10 production of T cells was evaluated using IL-10 secretion assay and CytoStim™ kits (Miltenyi Biotec); next, the cells were stained with anti-CD4, anti-CD49b, anti-LAG-3, anti-TIM-3, anti-TIGIT, anti-PD1, anti-CTLA-4, anti-CD39 and Zombie Aqua™ for 30 min at 37°C in the dark and washed once with FACS buffer. The chemokine profile was evaluated with anti-CD4, anti-CD49b, anti-LAG-3, anti-CCR2, anti-CCR5, anti-CCR4, anti-CCR7, anti-CXCR3, and Zombie NIR™ and the samples were stained for 20 min at room temperature in the dark and washed once with FACS buffer. Sample acquisitions were performed in an Attune NxT cytometer (Thermo Scientific) and data were analyzed with FlowJo v10.8 software (BD Biosciences, CA, USA).

### 2.7 High dimensional analysis of flow cytometry data

To capture the non-linear structure of our single cell data, we performed dimensionality reduction using a FlowJo implementation opt-tSNE (22). To identify clusters within the opt-tSNE map, X-Shift was performed as previously described (23). The X-Shift identified clusters were then phenotyped using FlowJo v10.8’s plug-in ClusterExplorer and ViolinBox (San Diego, BD).

### 2.8 *In vitro* allo-specific suppression assay

Autologous CD3^+^ responder T cells (R.T.C.) were isolated from PBMCs using the Pan T Cell Isolation Kit (Miltenyi Biotec) and labeled with CFSE, following the manufacturer’s instructions. Expanded allospecific Tr1 (^Exp-allo^ Tr1) cells expanded for three weeks were labeled with CTV, co-cultured with CFSE-labeled R.T.C. at different ratios CD3^+ Exp-allo^ Tr1 (1:1, 1:3, 1:9 and 1:27) and stimulated with specific-allogeneic or third-party moDC in a ratio 1:2 (CD3^+^:moDC) in expansion culture medium with 10% AB-HS. For some assays, co-cultures were stimulated in the presence or absence of 5 ng/mL of IFN-γ, IL-1β, IL-6 and/or TNF-α. After 4 days of co-culture, cells were recovered and stained with mAbs anti-CD3, anti-CD4 and anti-CD8, cells were acquired in an Attune NxT Cytometer and data were analyzed with FlowJo v10.8. software. The R.T.C. proliferation was determined by CFSE dilution on gated CD4^+^ or CD8^+^ T cells, and CTV-labeled ^Exp-allo^ Tr1 cells were excluded from the analysis. Results are presented as relative increments (RI) of the suppression obtained in control cultures without ^Exp-allo^ Tr1 cells. The relative increment was calculated using the following formula:


RI=%Proliferation of the sample/%Proliferation of R.T.C. without Tr1

### 2.9 Tr1 cells stability assays

After 3 weeks of expansion, ^Allo^ Tr1 cells were stimulated with 2 additional cycles of polyclonal expansion/resting in the presence or absence of rhIL-6 (5 ng/mL), rhTNF-α (5 ng/mL), rhIFN-γ (5 ng/mL) and/or IL-1β (5 ng/mL). Tr1 cell stability was evaluated by analyzing the expression of CD49b, LAG-3, IL-10, TIM-3, TIGIT, PD1, CTLA-4, CD39, as well as their alloantigen-specific suppressive function by flow cytometry.

### 2.10 Cytokine production assay

For cytokine production analysis, CD4^+^ T cells (^Exp-allo^ Tr1 and T conv) (2x10^4^ cells/well) were stimulated with anti-CD3/anti-CD28 beads (beads:T cells at a ratio of 1:5) during 4 days. The concentrations of cytokines in the culture supernatants were measured using the LEGENDplex™ kit, Custom Human Panel (Biolegend), according to the manufacturer’s guidelines. The samples were acquired on the flow cytometer CytoFLEX S (Beckman Coulter) and data were analyzed using FlowJo (BD) and GraphPad Prism v8 softwares. Cytokine concentrations were determined using the standard curve generated in the same assay.

### 2.11 Statistical analysis

Statistical analysis was performed using GraphPad Prism v8 software. The Shapiro–Wilk test was used to evaluate the distribution of the data. Differences between two groups were calculated using paired and unpaired t tests for normally distributed data; Wilcoxon’s rank sum test or Mann-Whitney test were used for non-normally distributed data. Graphs are expressed as Mean ± Standard Error of the Mean (SEM). Values with p<0.05 were considered as statistically significant.

## 3 Results

### 3.1 *In vitro*-generated allogeneic DC_10_ efficiently induce ^Allo^ Tr1 cells

With the overall aim of increasing the yield and purity of the cellular products obtained with current Tr1-based methodologies, we designed a new experimental protocol that allows efficient long-term expansion of highly purified ^Allo^ Tr1 cells (**Figure 1**). Based on previous reports (24), we used *in vitro* differentiated DC_10_ as an efficient methodology to the differentiation of ^Allo^ Tr1 cells.

**Figure 2:**
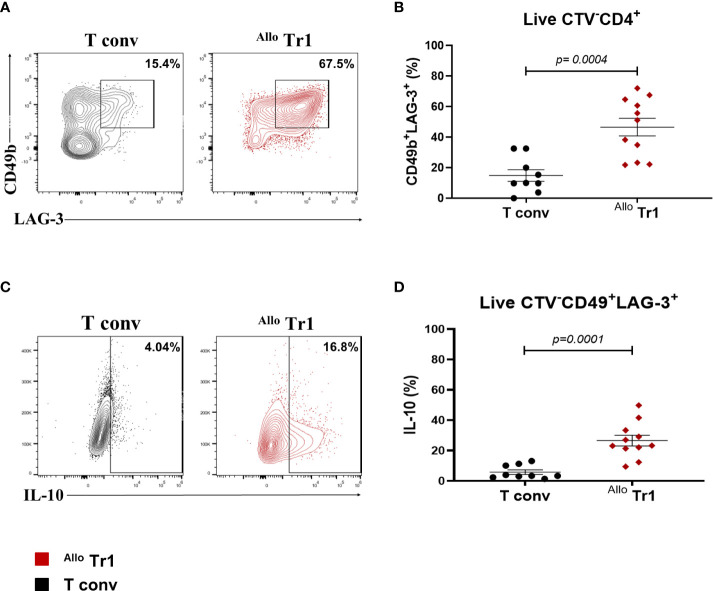
DC_10_ induces the differentiation of a high percentage of CD49b^+^LAG-3^+ Allo^ Tr1 cells. nT (CD4^+^CD25^-^CD45RA^+^) cells were co-cultured with allogeneic moDC (Allo T conv) or DC_10_ (^Allo^ Tr1 cells) for 14 days. **(A)** Representative contour-plot of CD49b and LAG-3 co-expression in proliferating cells from T conv (black) or ^Allo^ Tr1 cells (red). **(B)** Proportion of CD49b^+^LAG-3^+^ in ^Allo^ Tr1 (red, n= 11) condition culture compared to T conv (black, n= 9) condition. **(C)** Representative contour-plot of IL-10 production from CD49b^+^LAG-3^+^ population in T conv (black) and ^Allo^ Tr1 cells (red). **(D)** CD49b^+^LAG-3^+ Allo^ Tr1 cells (red, n= 11) showed a significantly higher IL-10 production compared with CD49b^+^LAG-3^+^ T conv (black, n= 9). All experiments were performed in duplicates. Data are representative of six independent experiments. The results are shown as mean ± SEM. The statistical analysis was performed using the unpaired-t test or the Mann–Whitney U test.

We first evaluated the phenotype of DC_10_, which presented a tolerogenic dendritic cell phenotype characterized by a significant increase of HLA-G^+^ILT4^+^ and CD14^+^ percentages compared to moDC (**Figure S1**). Next, CD4^+^CD25^-^CD45RA^+^ naïve T cells were sorted by FACS, CTV-labeled and co-cultured with allogeneic DC_10_ in the presence of rhIL-10 and rhIL-2 for 14 days and then analyzed the expression of specific Tr1 surface markers CD49b and LAG-3. As a negative control for Tr1 differentiation, nT cells were stimulated with moDC and only IL-2. We obtained a higher percentage of CD49b^+^LAG-3^+ Allo^ Tr1 cells in co-cultures stimulated with allogeneic DC_10_ compared to allogeneic moDC (46.5% ± 19.1 vs 14% ± 11) (**Figures 2A, B**). Moreover, ^Allo^ Tr1 cells differentiated with DC_10_ showed a significant increase in IL-10 production (28.7% ± 15 vs 6% ± 5) (**Figures 2C, D**). Using our culture conditions, we were able to differentiate a high percentage of ^Allo^ Tr1 cell population with Tr1 cell phenotype.

### 3.2 Long-term polyclonally-expanded Alo-Tr1 cells maintain an enriched suppressor phenotype and high IL-10 production

**Figure 3:**
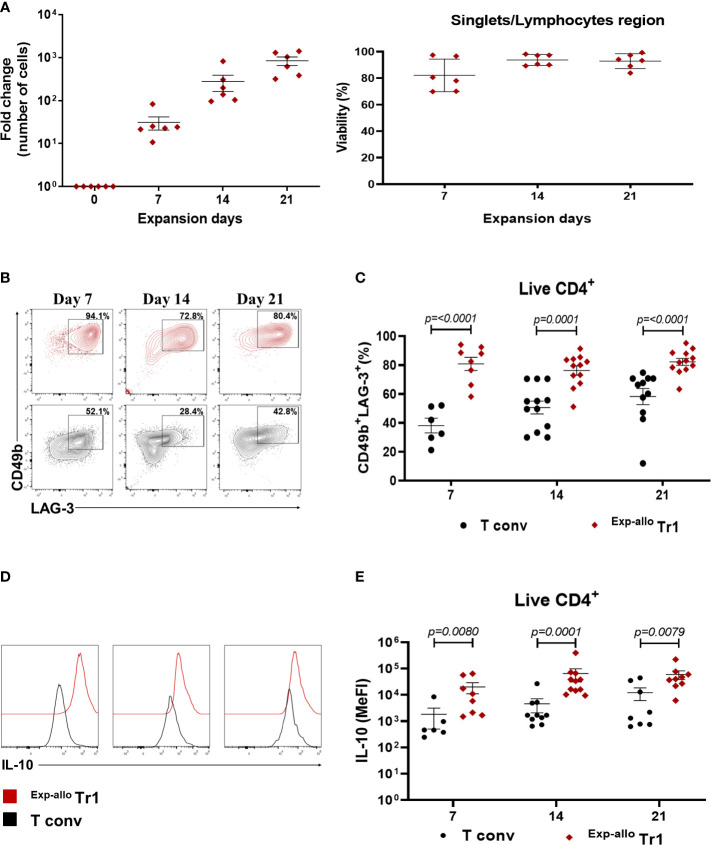
Purified ^Allo^ Tr1 cells can be long-term expanded, conserving their CD49b^+^LAG3^+^ phenotype and IL-10 production. FACS-sorter ^Allo^ Tr1 cells or T conv were polyclonally-expanded for 21 days in the presence of IL-10 plus IL-2 or only IL-2, respectively. **(A)** Left, **^A^** ^llo^ Tr1 cell proliferation reaching an average fold increase of 840 times at day 21 of stimulation (n= 6). Fold expansion was calculated by dividing the number of Tr1 cells obtained on the evaluated day by the number of Tr1 cells on day 1 of FACS-sorting. Right, viability of ^Exp-allo^ Tr1. Data are representative of four independent experiments. **(B)** Representative contour-plot of LAG-3 and CD49b co-expression in ^Exp-allo^ Tr1 cells (red) and T conv (black) at each cycle of *in vitro* expansion. **(C)** Proportion of CD49b^+^LAG-3^+^ in ^Exp-allo^ Tr1 cells (red, n= 6-12) compared to T conv (black, n= 6-12). **(D)** Representative histograms of IL-10 production in ^Exp-allo^ Tr1 cells and T conv. **(E)** Median Fluorescence Intensity (MeFI) of IL-10 in ^Exp-allo^ Tr1 cells (red) compared with T conv (black) throughout the expansion. All experiments were performed in duplicates. Data are representative of six independent experiments. The results are shown as mean ± SEM. The statistical analysis were performed using the unpaired-t test or the Mann–Whitney U test.

To increase the purity and ^Allo^ Tr1 cell numbers, we isolated CD49b^+^LAG-3^+ Allo^ Tr1 cells from co-cultures with DC_10_ by FACS and then we developed an efficient expansion protocol including cycles of polyclonal activation (4 days) followed by resting (3 days with only IL-2) for 21 days. As previously reported, Tregs cells express surface markers that are also present in highly activated T conv, therefore, we performed parallel cultures after purification of allogeneic T conv cells followed by polyclonal expansion under the similar stimulation conditions as ^Allo^ Tr1. As shown the **Figure 3**, the numbers of purified ^Allo^ Tr1 cells were significantly augmented, reaching an average fold increase of 840 times (**Figure 3A**). Moreover, after evaluating the phenotype of ^Exp-allo^ Tr1 cells at each polyclonal expansion cycle (days 7, 14 and 21), we observed a higher proportion of CD49b^+^LAG-3^+^ cells, compared with expanded T conv cells, (cycle I: 80.7% ± 12.9 vs 38.1 ± 12.4; cycle II: 76.2% ± 11.16 vs 50.5% 15.3; cycle III: 82.1% ± 8.4 vs 58.22% ± 18.4) (**Figures 3B, C**). Importantly, ^Exp-allo^ Tr1 cells maintained significantly increased levels of IL-10 compared with T conv cells at each cycle of expansion (**Figures 3D, E**). Interestingly, the Median Fluorescence Intensity (MeFI) of LAG-3 was significantly higher in ^Exp-allo^ Tr1 compared to expanded T conv in each expansion cycle, while we did not observe differences in the expression levels of CD49b between both populations throughout the expansion protocol (**Figures S2A, B**).

**Figure 4:**
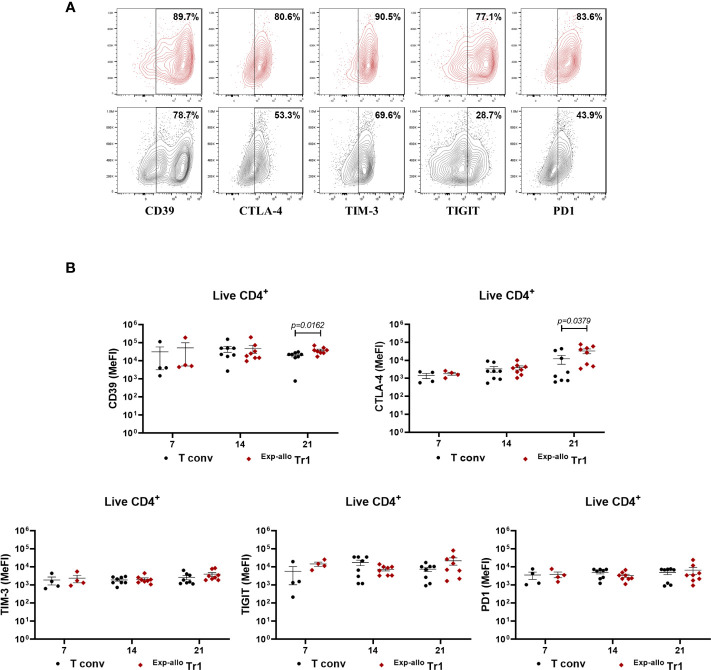
Long-term ^Exp-allo^ Tr1 cells maintain a high expression of co-inhibitory receptors. Purified ^Allo^ Tr1 cells were polyclonally expanded for 21 days and the expression of co-inhibitory receptors were evaluated. **(A)** Representative contour-plot of expression of co-inhibitory receptors in ^Exp-allo^ Tr1 cells (red) and Allo T conv (black). **(B)** Levels of expression (MeFI) of TIM-3, TIGIT, PD1, CTLA-4 and CD39 in ^Exp-allo^ Tr1 (red, n= 4-8) and Allo T conv (black, 4-8) at day 21 of stimulation. All experiments were performed in duplicates. Data are representative of six independent experiments. The results are shown as mean ± SEM. The statistical analysis were performed using an unpaired-t test or the Mann–Whitney U test.

To further investigate the phenotypic markers of ^Exp-allo^ Tr1 cells, we evaluated the expression of co-inhibitory receptors related to their suppressive function at 7, 14, and 21 days of polyclonal expansion. Importantly, both the percentage (**Figures 4A**, **S3A**) and expression levels (MeFi, **Figure 4B**) of TIM-3, TIGIT and PD-1 were maintained in ^Exp-allo^ Tr1 cells throughout the polyclonal expansion. Of note, these co-inhibitory receptors were similarly expressed in expanded T conv, as it would be expected in long-term stimulated T cells (**Figures S3A, B**). Interestingly, ^Exp-allo^ Tr1 cells showed significantly increased levels (MeFI) of CD39 and CTLA-4 (**Figure 4B** **)** at 21 days of expansion compared to expanded T conv cells.

### 3.3 High dimensional analysis of ^Exp-allo^ Tr1 confirms a distinct regulatory profile that differentiates from expanded T conv cells

**Figure 5:**
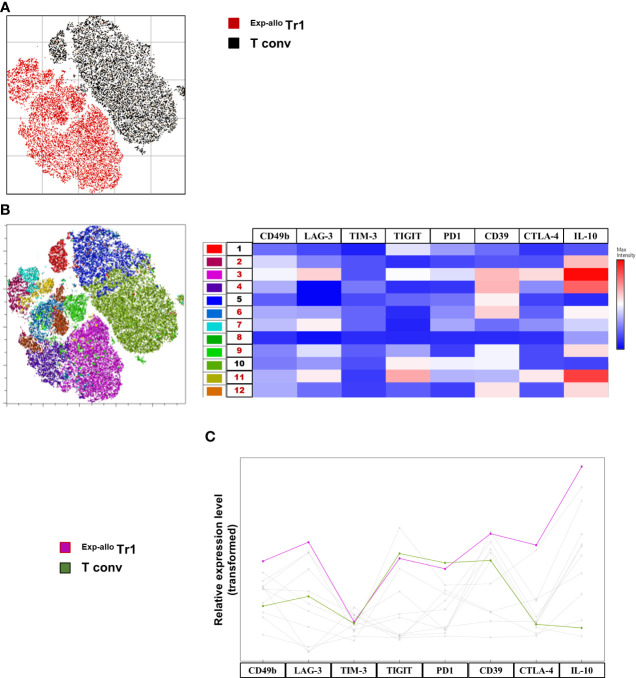
High dimensional analysis of ^Exp-allo^ Tr1 shows a differential expression profile compared to T conv cells. Purified ^Allo^ Tr1 cells were polyclonally expanded for 21 days, co-inhibitory receptors were evaluated, and high dimensional analysis was used to further identify and explore their expression profile. **(A)** Dimensionality reduction by Opt-SNE from live CD4^+ Exp-allo^ Tr1 (n= 3) cells and T conv (n= 3) cells showed two major islands characterized by Tr1 (red) versus T conv (black) culturing conditions right panel). **(B)** X-Shift clustering analysis identified 11 clusters (excluding cluster 8, which represents 0.18% of the Tr1 population) corresponding to one major island composed of ^Exp-allo^ Tr1 (2, 3, 4, 6, 7, 9, 11 and 12) and the other with T conv (1, 5 and 10). The resulting heat map of median fluorescence expression from each cluster shows a distinct expression profile between ^Exp-allo^ Tr1 and T conv clusters. Clusters 3 and 10 were selected as representative of each culturing conditions clearly showing overexpression IL-10, CD39, LAG-3 and CTLA-4 wherein T conv were relatively low expressors for all these markers compared to ^Exp-allo^ Tr1 **(C)**. Relative expression line graph of median fluorescence intensity shows the level expression of each co-inhibitory molecule from the underlying most representative populations: ^Exp-allo^ Tr1 (pink) vs T conv (green). Data are representative of two independent experiments.

Opt-tSNE was performed on live CD4^+^ cells from individual samples were down-sampled to 12,660 events per sample, individual samples were electronically barcoded, and finally concatenated for downstream analyses. A total of six samples post-expansion (from three individuals) were included in the analysis, three ^Exp-allo^ Tr1 cells and the corresponding T conv cells. Opt-tSNE was run using all compensated parameters except the previously gated live CD4 and prior parent populations. Two major islands were identified in the resulting opt-tSNE maps. These were pulled together predominantly according to ^Exp-allo^ Tr1 versus T conv expanded populations. Both major islands contained populations from all three patients (**Figure 5A**), demonstrating a non-patient bias in the dimensionality reduction.

To identify cell clustering within our high-dimensional data visualized with opt-tSNE, we ran X-Shift on the live CD4^+^ population (23). X-shift clustering identified 11 clusters (**Figure S4**), three clusters (Clusters 1, 5, 10) were found to have been composed exclusively from T conv cells and composing one of the two major opt-tSNE islands (**Figure 5B**). The remaining eight clusters (Clusters 2-4, 6-8, 9, 11-12) were found to have been composed exclusively of the ^Exp-allo^ Tr1 cells and composing the other major opt-tSNE island (**Figure 5B**). FlowJo’s ClusterExplorer and ViolinBox were used to phenotype the X-Shift clusters (**Figure 5C**). Importantly, only the eight clusters identified in the expanded Tr1 cells (Clusters 2-4, 6-8, 9, 11-12) expressed high IL-10, albeit at varying levels of expression. In contrast, T conv cell related clusters expressed lower levels of IL-10. We selected clusters 3 and 10, as the most representative of ^Exp-allo^ Tr1 (46%) and T conv (70%) respectively, and compared their expression profiles. As shown in **Figures 5B, C**, Cluster 3, in addition to IL-10, showed distinctive high levels of CD49b, CD39, CTLA-4 and LAG3 compared to Cluster 10, while both clusters expressed similar levels of TIGIT, TIM-3 and PD-1.

### 3.4 ^Exp-allo^ Tr1 cells express a chemokine receptor profile relevant for solid organ allotransplantation homing

**Figure 6:**
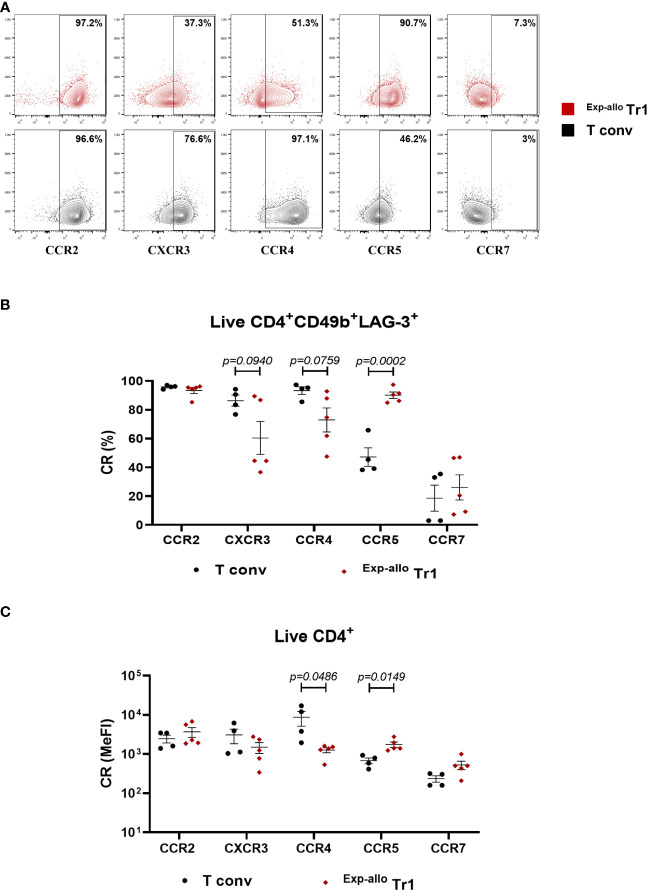
^Exp-allo^ Tr1 cells show a high expression of chemokines receptors relevant for homing to allografts. Purified ^Allo^ Tr1 cells were polyclonally expanded for 21 days and the expression of CR receptors were evaluated. **(A)** Representative contour-plot of chemokines receptors expression in ^Exp-allo^ Tr1 cells (red) and Allo T conv (black). **(B)** Percentages or **(C)** levels of expression (MeFI) of chemokine receptors CCR2, CXCR3, CCR4, CCR5 and CCR7 in ^Exp-allo^ Tr1 cells (red, n= 5) and T conv (black, n= 4) at day 21 of expansion. All experiments were performed in duplicates. Data are representative of three independent experiments. The results are shown as mean ± SEM. The statistical analysis was performed using the unpaired t-test or the Mann–Whitney U test.

The phenotypical characterization of Tr1 cell populations candidates in cellular therapy, has mainly focused on the expression of molecules involved in their suppressive function, nevertheless, the evaluation of CR expression is key to ensure the migratory potential of infused Tr1 and the induction of effective tolerance towards de graft. To evaluate the homing potential of our ^Exp-allo^ Tr1 cells, we evaluated at day 21 of expansion the expression of CCR2, CXCR3, CCR4, CCR5 and CCR7 which are known to direct the migration of T cells to the allograft and draining lymph nodes, respectively (**Figure 6**). As previously reported for Tr1 cells (17), we showed a high percentage of ^Exp-allo^ Tr1 cells expressing CCR5 (90.09% ± 4.9) and this was significantly increased compared to T conv cells (47.1% ± 12.8%). On the other hand, expression of CXCR3 and CCR4 showed a trend towards a decrease in ^Exp-allo^ Tr1 cells compared to T conv cells (CXCR3 60.3% ± 25.51 vs 86.30% ± 7.7, *p=0.0940*; CCR4 60.3% ± 9.7 vs 93.2% ± 5.2, *p=0.0759*). By contrast, we found lower expression of CCR7 in both ^Exp-allo^ Tr1 cells (26.08% ± 19.4) and T conv cells (18.6% ± 18) compared to other receptors while high proportion of both populations were CCR2^+^ (93.3% ± 4 vs 95.8% ± 1.1) (**Figure 6B**). Interestingly, when we compared the MeFI values of CR expression, we found significantly increased levels of CCR5, but decreased levels of CCR4, in ^Exp-allo^ Tr1 cells compared to T conv cells. Finally, CXCR3 was moderately upregulated on T conv compared to ^Exp-allo^ Tr1 cells and no significant differences were found in CCR2 and CCR7 MeFI values between both subpopulations (**Figure 6C**).

### 3.5 ^Exp-allo^ Tr1 cells secrete cytokines with an anti-inflammatory profile

**Figure 7:**
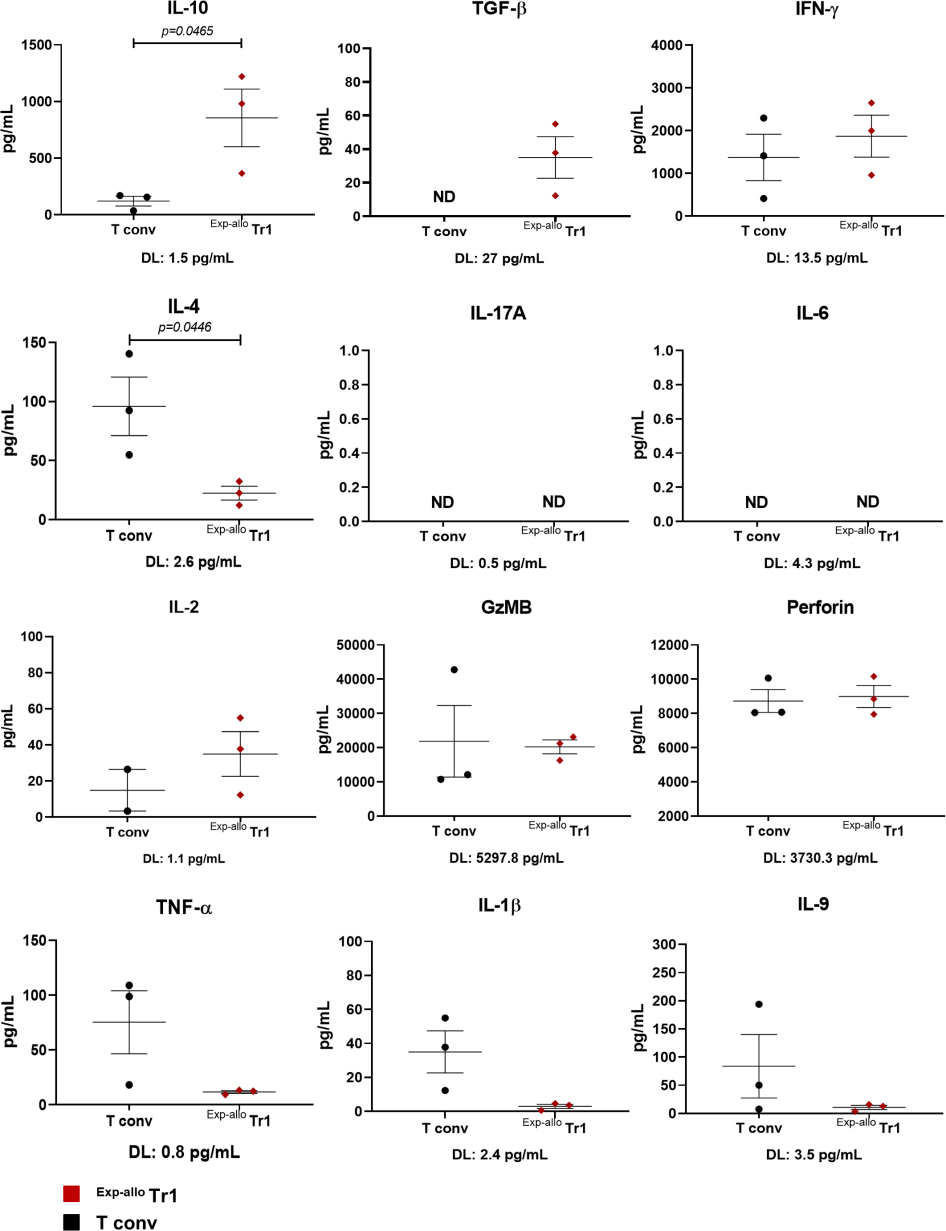
Prolonged expanded ^Allo^ Tr1 has a higher production of anti-inflammatory cytokines, but does not produce inflammatory cytokines. ^Exp-allo^ Tr1 cells (red, n= 3) and T conv (black, n= 3) cells expanded for three weeks were restimulated for 4 days, and supernatants were analyzed for cytokine production by cytometric bead array. The detection limits (pg/mL) for each cytokine are shown below each graph. All experiments were performed in duplicates. Data are representative of three independent experiments. The results are shown as mean ± SEM (n= 3). Statistical analysis was performed using an unpaired t-test or the Mann Whitney U test.

Another essential aspect to consider in the characterization of Tr1 cells is their cytokine production profile. We evaluated the cytokine secretion from ^Exp-allo^ Tr1 cell supernatant cultures, using a cytokine-based assay (CBA) (**Figure 7**). As we expected, the production of the anti-inflammatory cytokines IL-10 and TGF-β were significantly higher in ^Exp-allo^ Tr1 cultures compared to expanded T conv cells. By contrast, the production of pro-inflammatory cytokines TNF-α, and IL-1β and IL-4, by ^Exp-allo^ Tr1 was decreased when compared to T conv cells. Additionally, the production of IL-17, IL-6, and IL-9 were very low or undetectable in both T cell populations. The secretion of IFN-γ, granzyme B (GzMB) and perforin has been previously reported in Tr1 cells (reviewed in (7)); here, we found a similar production of GzMB and perforin but a trend towards to increase the IFN-γ production in ^Exp-allo^ Tr1 compared to T conv cells. Remarkably, the production of IL-2 was lower in T conv in comparison with ^Exp-allo^ Tr1. In conclusion, the cytokine production of the ^Exp-allo^ Tr1 cells fits with an anti-inflammatory profile.

### 3.6 ^Exp-allo^ Tr1 cells suppress responder T cells proliferation efficiently

**Figure 8:**
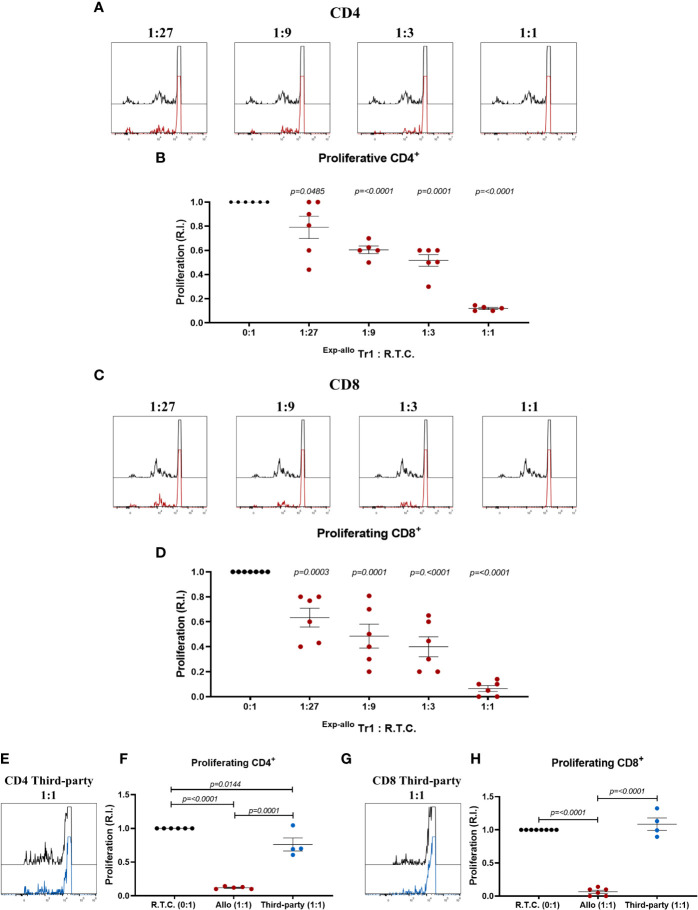
*In vitro* ^Exp-allo^ Tr1 cells suppress the proliferation of T conv cells in an alloantigen-specific way. ^Exp-allo^ Tr1 were co-cultured with conventional CD3^+^ R.T.C. (labeled with CFSE) at different evaluated ratios and stimulated with allogeneic moDC from their respective donors or from non-related individuals (Third Party); on day 4 of culture, R.T.C. proliferation was evaluated by flow cytometry. **(A–D)** ^Exp-allo^ Tr1 inhibited the proliferation of both CD4^+^ **(A, B)** and CD8^+^ **(C, D)** R.T.C. at all evaluated ratios (n= 5-6). **(E–H)** ^Exp-allo^ Tr1 suppresses the proliferation of CD8^+^ **(E, F)** and CD4^+^ **(G, H)** R.T.C. cells only when they are stimulated with the allogeneic DCs with which they were initially expanded (Allogenic, red, n= 4-5), but do not suppress when they are stimulated with unrelated DCs (Third Party, blue, n= 4). Representative histograms are shown in **(A)**, **(C)** and **(E)**. All experiments were performed in duplicates. Data are representative of four independent experiments. The results are shown as mean ± SEM. Statistical analysis was performed using the unpaired-t test or one sample t and Wilcoxon test.

After phenotypical characterization of ^Exp-allo^ Tr1 cells and to ensure the functionality, we next evaluated their ability to suppress allospecifically CD4^+^ and CD8^+^ responder T cells (R.T.C.). As shown in **Figure 8**, we observed a reduction of the R.T.C. proliferation at all ratios evaluated, showing the most significant suppression at the 1:1 ratio for both CD4^+^ (**Figures 8A, B**) and CD8^+^ T cells (**Figures 8C, D**). Importantly, the suppressive capacity of ^Exp-allo^ Tr1 cells was significantly lower when T cells were stimulated with allogeneic moDC from a not related individual (Third-party) compared to the DCs towards which they were initially expanded (Allo) for both CD4^+^ (**Figures 8E, F**) and CD8^+^ (**Figures 8G, H**) proliferation.

**Figure 9:**
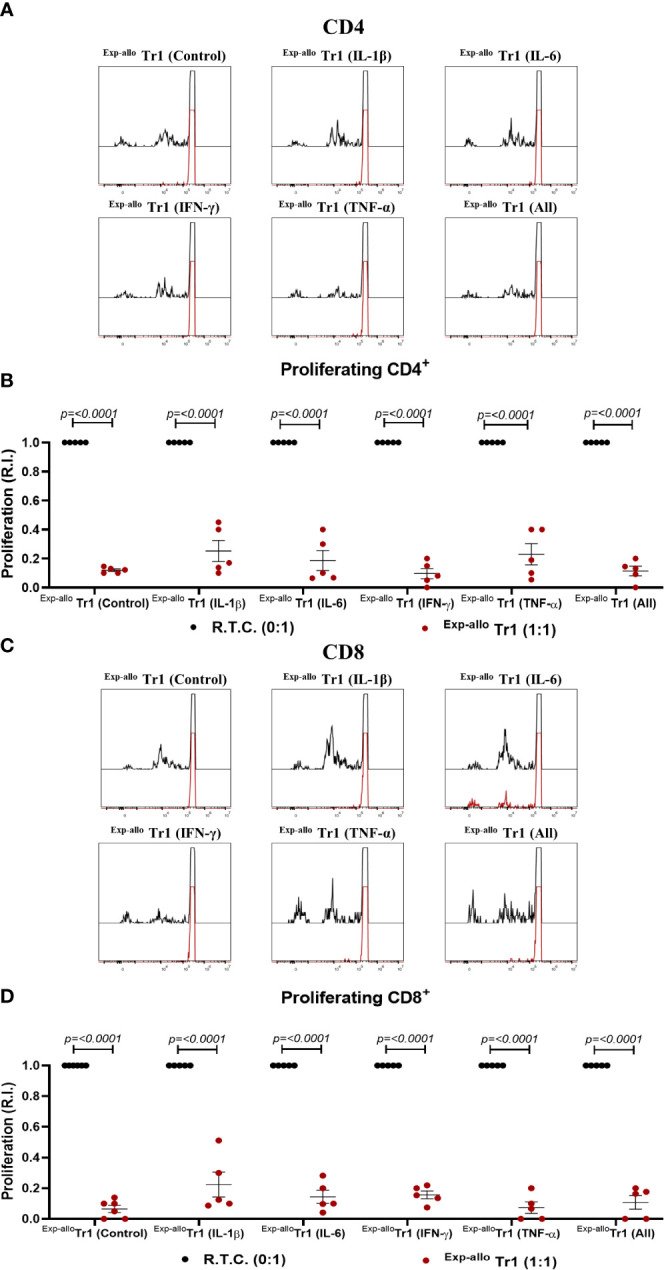
^Exp-allo^ Tr1 cells maintain their suppressive capacity in presence of inflammatory cytokines. Co-culture of ^Exp-allo^ Tr1 and conventional CD3^+^ T cells were stimulated with allogeneic moDC in the presence or absence of inflammatory cytokines and T cell proliferation was evaluated. **(A–D)** ^Exp-allo^ Tr1 efficiently suppressed the proliferation of both CD8^+^ **(A, B)** and CD4^+^ **(C, D)** R.T.C. in the presence of inflammatory cytokines IL-1β, IL-6, IFN-γ, TNF-α or all together (n= 5). All experiments were performed in duplicates. Data are representative of four independent experiments. The results are shown as mean ± SEM. Statistical analysis was performed using the unpaired-t test or one sample t and Wilcoxon test.

Under inflammatory conditions, it has been demonstrated that some cytokines (IL-1β, TNF-α, IL-6) may be involved in FOXP3^+^ Treg suppression downmodulation (18, 25, 26), however this has not yet been extensively studied in Tr1 populations. Thus, we evaluated the suppression of ^Exp-allo^ Tr1 cells in the presence of an inflammatory environment. Markedly, ^Exp-allo^ Tr1 cells were able to suppress the CD4^+^ (**Figures 9A, B**) and CD8^+^ (**Figures 9C, D**) R.T.C proliferation even in the presence of exogenous IL-1β, IL-6, IFN-γ and TNF-α in the co-cultures, either alone or all together.

### 3.7 Proinflammatory cytokines do not affect the phenotype and suppressive function in ^Exp-allo^ Tr1 cells

**Figure 10:**
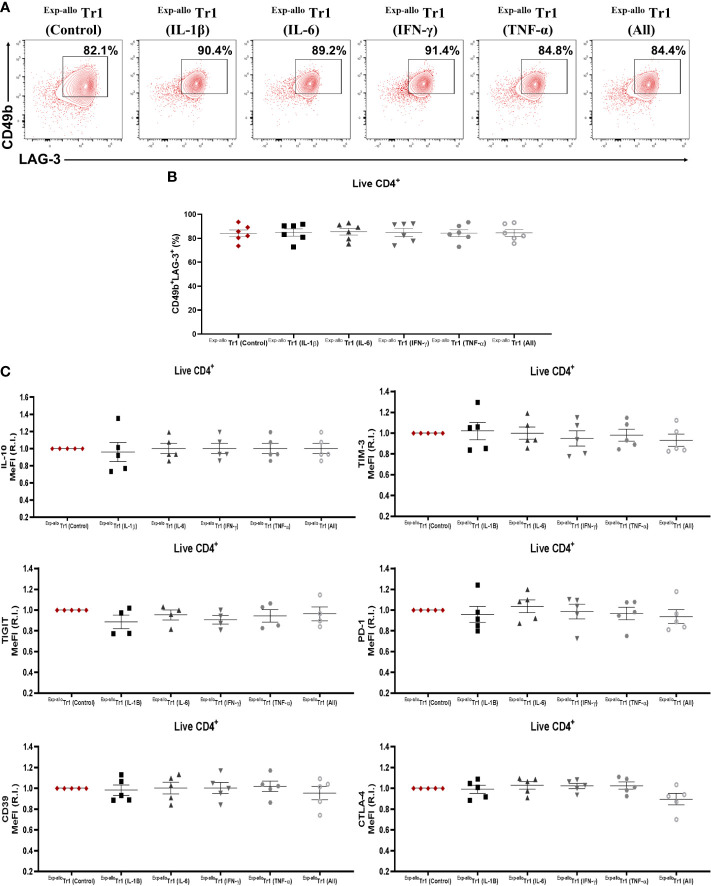
^Exp-allo^ Tr1 cells maintain their immunosuppressive phenotype after stimulation in an inflammatory microenvironment. ^Exp-allo^ Tr1 cells were stimulated with anti-CD3/anti-CD28 in the presence or absence of pro-inflammatory cytokines (IFN-γ, IL-6, IL-1β, TNF-α or all together) for two additional weeks. **(A)** Representative contour-plot of CD49b and LAG-3 expression in ^Exp-allo^ Tr1 activated in presence of inflammatory cytokines. **(B)** Proportion of CD49b^+^LAG-3^+^ expression in ^Exp-allo^ Tr1 cells activated in presence or absence of inflammatory cytokines (n= 5). **(C)** Relative increase (IR) of IL-10, TIM-3, TIGIT, PD-1, CD39 and CTLA-4 (MeFI). RI was calculated by dividing the value of all the conditions by the value of control ^Exp-allo^ Tr1 condition. All experiments were performed in duplicates. Data are representative of four independent experiments. The results are shown as mean ± SEM. Statistical analysis was performed using the unpaired-t test or one sample t and Wilcoxon test. No significant differences were observed.

It has been widely reported that an inflammatory microenvironment can directly influence the phenotype or functionality of CD4^+^ T cells. Thus, ^Exp-allo^ Tr1 cells were further expanded for two extra weeks in the presence of IL-2 plus rhIL-1β, rhIL-6, rhIFN-γ, rhTNF-α, or all together, or with IL-10 (^Exp-allo^ Tr1 control conditions). After these two extra expansion rounds, we did not observe significant difference in neither CD49b and LAG-3 (**Figures 10A, B**) nor in TIM-3, TIGIT, PD-1, CD39, CTLA-4 expression and in the IL-10 production under proinflammatory cytokine conditions (**Figure 10C**).

**Figure 11:**
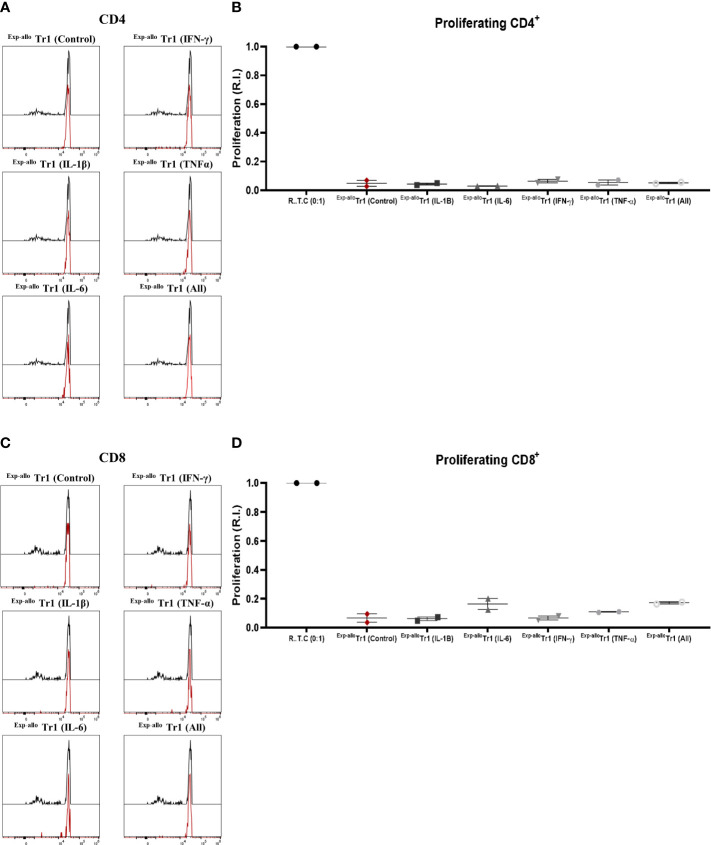
^Exp-allo^ Tr1 cells maintain their suppressive function after stimulation in an inflammatory microenvironment. ^Exp-allo^ Tr1 activated in presence or absence of pro-inflammatory cytokines (IFN-γ, IL-6, IL-1β, TNF-α or all together) were co-cultured with conventional CD3^+^ R.T.C. (labeled with CFSE) at 1:1 ratio and stimulated with allogeneic moDC from their respective donors, on day 4 of co-culture. **(A–D)** ^Exp-allo^ Tr1 efficiently suppressed the proliferation of both CD4^+^ **(A, B)** and CD8^+^ **(C, D)** R.T.C. after being activated in presence of IL-1β, IL-6, IFN-γ, TNF-α or all together (n= 2). All experiments were performed in duplicates. Data are representative of two independent experiments. The results are shown as mean ± SEM. Statistical analysis was performed using the unpaired-t test or one sample t and Wilcoxon test.

Once we demonstrated that phenotype and IL-10 production were not affected by pro-inflammatory cytokines, we next evaluated if ^Exp-allo^ Tr1 cells were able to maintain the capacity to suppress R.T.C. proliferation. As shown in **Figure 11**, we observed that ^Exp-allo^ Tr1 preserved their suppressive potential towards CD4 and CD8 R.T.C. proliferation, even after being expanded in the presence of all the pro-inflammatory cytokines together. (**Figures 11A, B**). These results support our previous observation of the stability of ^Exp-allo^ Tr1 suppressive function in presence of proinflammatory cytokines (**Figure 8**).

## 4 Discussion

In recent years new advances in the *in vitro* generation of Tr1 have demonstrated their potential as therapeutic tools for transplantation tolerance. IL-10 tolerogenic capacity was exploited to establish two experimental protocols; one used allogeneic CD3-depleted PBMC to generate IL-10 anergized T cells, containing Tr1 cells (IL-10-DLI) (27), and the other used tolerogenic DC_10_, with high production of IL-10 and co-expression HLA-G and ILT-4 (24), giving rise to T10 or T-allo 10 (reviewed in (7). Both methodologies have been implemented using GMP grade standards showing promising results in clinical trials and a beneficial outcome in HSCT patients for improving immune reconstitution without increasing the risk of GvHD (ALT-TEN (15), NCT01656135 (16), and NCT03198234 (17).

As previously mentioned, we used monocyte-differentiated DC_10_ in ours protocol and we were able to generate a higher percentage of CD49b^+^LAG-3^+ Allo^ Tr1 compared to previous works (46.5% ± 19.1 vs 6% ± 3 (16) and 10.5% [9 to 13% interquartile range (IQR)] (17). These differences might be because we performed some modifications in the original protocol, including a restimulation with rhIL-10 (10 ng/mL) and rhIL-2 (50 U/mL) in the co-cultures at day 7, compared to the work of Gregory et al, where only add rhIL–2 (20 U/mL) (24). It has been reported that IL-10 signaling is required to promote Tr1 cell differentiation (28), while IL-2 signaling promotes the proliferation and survival of T cells (29). Additionally, we used non-irradiated DC_10_ as stimulators which were able to induce a higher percentage of CD49b^+^LAG-3^+ Allo^ Tr1 compared to irradiated DC_10_ (46.5% vs 12.0%, data not shown). These results suggest that in addition to IL-10 production and HLA-G/ILT4 signaling (24), another important stimulus provided by non-irradiated DC_10_ could be contributing to the ^Allo^ Tr1 differentiation.

When we evaluated IL-10 production by a flow cytometry-based IL-10 secretion assay at day 14 of co-culture (DC_10_:nT), we found an average of 28.7% ± 15 of IL-10^+^ cells within the CD49b^+^LAG-3^+^ population, which identifies functional Tr1 cells. This method ensures a direct detection of IL-10 producing ^Allo^ Tr1 cells (9), without interference of other potential contaminating subpopulations in the co-culture, which cannot be avoided in the studies using ELISA-based methods previously reported (16, 17).

One of the main challenges in the field of Tr1 therapy is the isolation of a pure Tr1 cell population that could increase the efficiency of the treatment *in vivo* and reduce possible long-term side effects induced by contaminating effector cells in the final population. Additionally, cell therapy using FACS-based isolation protocols have shown safety in clinical trials in kidney transplant patients (30, 31). With this aim in mind, we purified the CD49b^+^LAG-3^+^ cells from the initial allogeneic co-cultures by FACS sorting to ensure the obtention of an homogeneous Tr1 cell population. This is highly relevant for cell therapy purposes as it has been recently reported that Tr1 cells are the main functionally active component within T-allo 10 cell product displaying different suppressive mechanisms (17). Thus, infusion of a highly purified Tr1 subpopulation may allow to reduce the total cell numbers required to maintain long term tolerance.

To date, few clinical trials have been reported using a heterogeneous population of tolerogenic cells that contain different proportions of Tr1 cells. These studies determined the number of cells to be infused per kg of body weight based on the characteristics of the cellular population. It has been suggested that the number of cells required for clinical trials ranges from 3 x 10^5-6^/kg (CD3^+^ cells) (15) to 11-19 x 10^6^/kg containing sufficient Tr1 cells for inducing efficient allograft tolerance. However, in these studies the actual numbers of Tr1 cells was not directly evaluated, but rather, the numbers of infused cells were adjusted based on their *in vitro* tolerogenic potential (16). Considering the relatively low numbers of purified Tr1 cells obtained from our allogeneic co-cultures, we developed a polyclonal expansion protocol that allowed an increase of 840 times the initial numbers at day 21. In addition, our protocol allows us to determine the numbers of Tr1 cells transferred and may reduce the numbers Tr1 needed to achieve tolerance. To our knowledge this is the first protocol that enables allospecific Tr1 cell expansion. A different approach for total polyclonal Tr1 *ex vivo* expansion has recently reported a 70-fold increase after 18 days of expansion with anti-CD3 and anti-CD28 beads (32), although the authors reported less than 50% of CD49b^+^LAG-3^+^ cells within the expanded population, while our expansion protocol maintained more than 80% of ^Allo^ Tr1 for over three weeks of expansion.

Currently, co-expression of CD49b and LAG-3 molecules together with IL-10 production, are the current markers used to identify Tr1 cells. Interestingly, ^Exp-allo^ Tr1 cells showed higher expression levels of LAG-3 compared to T conv cells and furthermore, IL-10 production was maintained through the expansion cycles **(** **Figure 2**), demonstrating that LAG-3 and IL-10 are closely related to ^Exp-allo^ Tr1. In addition, the expression of other co-inhibitory molecules have also been related in different scenarios to the Tr1 cell identity as well as their capacity to suppress effector T cells. In this context, murine CD49b^+^LAG-3^+^IL-10^+^ Tr1 cells expressing TIM-3, TIGIT and PD-1 have been related with a higher suppressive function compared with “Tr1-like” subpopulations, showing low or null expression of these molecules (9). Another report demonstrated that the ectoenzyme CD39 is important for Tr1 suppressive functions through the generation of adenosine (33). While human Tr1 cells have shown a higher expression of CTLA-4 and PD-1 compared to non-Tr1 cells, moreover, both molecules were key for the suppressive function against effector cells (17). The constant expression of TIM-3, TIGIT, PD1, CD39, and CTLA-4 during 21 days of expansion on ^Exp-allo^ Tr1 was in agreement with a high suppressive function. Notably, the higher expression of CTLA-4, CD39, LAG-3 and IL-10 in our ^Exp-allo^ Tr1 at day 21, compared to T conv, might be directly related to their suppressive potential.

In addition to expressing co-inhibitory receptors, CIR^+^ Tr1 were also reported to express CCR5, which is also displayed by our ^Exp-allo^ Tr1. The functional relevance of CCR5 and CXCR3 expression on T conv cells has been described to be pathogenic in mice and human solid organ transplantation (34). In accordance, combined CXCR3 and CCR5 blockade were effective for prolonging allograft in a model of allogeneic heart transplantation (35), thus the expressión of these receptors in Tregs could suggest a tolerance induction as a result of direct suppression on T conv. However, detailed expression of chemokine receptors on *in vitro* differentiated Tr1 cells had not been yet evaluated. To aim for the potential clinical use of ^Exp-allo^ Tr1 cells to generate allograft tolerance, the evaluation of chemokine receptors with homing potential toward the allograft needs to be considered. In the context of kidney transplantation, expression of CXCR3, CCR2 and CCR4 on Foxp3^+^ Tregs was found to be key in preventing allograft rejection (reviewed in (20)). Therefore, expression of CXCR3, CCR2 and CCR4 in our ^Exp-allo^ Tr1 cells (**Figure 6**) strongly suggest their potential homing towards the kidney allograft.

As several co-inhibitor receptors are expressed by other T cell subpopulations, including long-term stimulated T cells, it was important a deeper phenotypic analysis that allowed us to distinguish our ^Exp-allo^ Tr1 cells from activated T conv cells, that may also display markers typically expressed by exhausted T cells (36, 37). In this context, high dimensional analysis of multiparametric flow cytometry data has been employed for the characterization of tumor-infiltrating lymphoid populations (38) as well as in phenotypic analysis of Treg cell subpopulations (39). Importantly, our opt-tSNE analysis demonstrated that ^Exp-allo^ Tr1 cells are clearly distinguishable from activated T conv cells, as they each group into distinct islands (**Figure 5A**) and with each island being differentiated by the expression of IL-10 (**Figures 5B, C**).

An important mechanism of Treg-mediated suppression is closely related to their cytokine secretion profile. As expected, and as previously reported in Tr1 cells, IL-10, TGF-β, IFN-γ, GzMB and perforin were significantly produced by our ^Exp-allo^ Tr1 confirming their identity (**Figure 7**). Besides, our ^Exp-allo^ Tr1 cells also produced low levels of IL-2 and IL-4 (8, 40, 41). On the other hand, parallel cultures using expanded T conv cells showed moderate production of IL-2 and IFN-γ which is is in agreement with the long-term expansion conditions used and correlate with the expression of several markers typically expressed by exhausted T cells (TIGIT, TIM-3 and PD-1) (29, 37). The secretion of IL-10 and TGF-β was higher in our ^Exp-allo^ Tr1 cells compared to T conv cells, according to their suppressive phenotype. In contrast, the pro-inflammatory cytokines TNF-α and the Th2 cytokine IL-4 were singularly produced by expanded T conv cells compared with ^Exp-allo^ Tr1 cells confirming their differential inflammatory versus regulatory phenotype. Of note, ^Exp-allo^ Tr1 cells did not produce detectable levels of IL-17 as it has been reported for effector T cells (42). In addition, GzMB and perforin, that are important mechanisms for Tr1-mediated suppressive function, were significantly expressed to comparable levels with activated T conv cells (9, 43, 44), indicating their cytotoxic potential.

Most impressive are the findings of the suppressive function of ^Exp-allo^ Tr1 cells against allospecific CD4^+^ and CD8^+^ T cells, in accordance with the phenotypic characteristics of these cells. In contrast with other reports where allogeneic suppression was assessed at a ratio of 1:1, using an heterogeneous population of Tr1-containing cells (16, 17), we explored the ^Exp-allo^ Tr1 suppressive potential by comparing different Tr1:R.T.C. ratios, showing that ^Exp-allo^ Tr1 are able to suppress both CD4^+^ and CD8^+^ allogeneic T cells from 1:27 ratio, showing the highest potential at 1:1 ratio. This allows us to identify more precisely the suppressive function of a specific number of Tr1, and therefore extrapolate the effective Tr1 dose needed for *in vivo* tolerance. Remarkably, after polyclonal expansion our ^Exp-allo^ Tr1 remains allospecific, as proliferation towards third-party is significantly lower than to allo DCs.

One of the major concerns in Treg therapy is to ensure long term stability of the infused cells (45). Several studies have reported that several cytokines, including IL-6, IL-23 TNF-α, IL-17 and IL-4, can promote a downregulation of FOXP3, leading to the loss of their suppressive capacity and acquisition of an inflammatory phenotype (18, 19). Importantly, our group has recently reported that purified allospecific FOXP3^+^ Tregs and *de novo* generated CD4^+^CD25^+^FOXP3^+^ allospecific iTregs cells can be efficiently expanded *in vitro* maintaining their phenotype and antigen-specific suppressive function, even under a proinflammatory environment (IFN-γ, IL-4, TNF-α and IL-6) (46, 47). However, it was not clear whether *in vitro* differentiated ^Allo^ Tr1 cells were able to maintain their phenotype and function under proinflammatory conditions. Moreover, an extensive profile of pro-inflammatory cytokines has been detected in the serum of transplanted patients and in animal models (48, 49). Exceptionally, our findings indicate high stability of ^Exp-allo^ Tr1 both in their phenotypic markers (CD49b, LAG-3, IL-10, TIM-3, TIGIT, PD1, CD39, and, CTLA-4) as well as in the maintenance of the allogeneic suppressive function, after two expansion cycles in presence of proinflammatory cytokines IL-1β, IL-6, IFN-γ, and TNF-α. This is relevant since pro-inflammatory cytokines have been detected in serum of transplanted patients and animal models (48, 49).

On the other hand, the analysis of Tr1 *in vivo* stability in a mouse model of allogeneic pancreatic islet transplantation demonstrated that transferred congenic Tr1 cells expand and can be traced more than 3 months after transplantation and are sufficient to induce long-term tolerance (50). Currently, it is hard to trace human transferred Tr1 cells, however, some reports suggest that *in vivo* Tr1 cells may be stable and promote the generation of new Tr1 cells (infectious tolerance) (51). Following this concept, we wondered if long-term *in vitro* expansion of ^Exp-allo^ Tr1 in a pro-inflammatory environment could be negatively affected.

Among the issues that remain to be evaluated are the molecular mechanisms involved in ^Exp-allo^ Tr1 stability, including analysis of single cell transcriptomics which would allow us to identify the key molecules (transcription factors, epigenetic mechanisms) that are key for the establishment and maintenance of the Tr1 lineage. In this context, different transcription factors have been reported to regulate Tr1-like phenotype and function (c-Maf, BATF, Blimp-1, Eomes, AhR, Erg2, IRF1) (8, 52), however more extensive studies are needed in human cells. We believe that our ^Exp-allo^ Tr1 provides a good model to explore these mechanisms, as it achieves the obtention of large numbers of purified antigen-specific Tr1 cells, which could also be used for genetic manipulations, such as those proposed for improving stability of Foxp3^+^ Tregs (53, 54) aiming to ensure long term stability *in vivo* in cell therapy protocols used both in transplantation as other immune-associated diseases.

One interesting issue that has not yet been addressed is the potential relationship between Foxp3^+^ Tregs and Tr1. The relatively high abundance and co-localization of these Treg subpopulations in some tissues, such as the small and large intestine (55), as well as reports showing the induction of Tr1 cells after Tregs transfer in a mice model of pancreatic islet transplantation (56) suggest a potential interplay between these two subpopulations that could be considered in future human clinical trials.

In summary, our data demonstrate for the first time the feasibility of expanding a purified allospecific Tr1 population efficiently and at a large-scale, maintaining their characteristic phenotype (CD49b^+^LAG-3^+^) and IL-10 production. Moreover, ^Exp-allo^ Tr1 cells express an enriched suppressive phenotype TIM-3, TIGIT, PD-1, CD39 and CTLA-4, and produce significant levels of TGF-β, IFN-γ, GzMB and perforin which correlate with their ability of suppress CD4^+^ and CD8^+^ proliferation in an alloantigen-specific manner. Most importantly, ^Exp-allo^ Tr1 are functionally stable even in the presence of inflammatory cytokines including IL-1β, IL-6, IFN-γ, TNF-α. We propose that this highly purified Tr1 population could improve efficiency *in vivo* of Tr1-based immunotherapy and reduce the risk of potential side effects produced by heterogeneous populations in long term organ transplanted patients.

## Data availability statement

The raw data supporting the conclusions of this article will be made available by the authors, without undue reservation.

## Ethics statement

The studies involving human participants were reviewed and approved by The Comité de Ëtica en Investigación" from the National Institute for Medical Sciences and Nutrition Salvador Zubirán, Reference 3886. Written informed consent for participation was not required for this study in accordance with the national legislation and the institutional requirements.

## Author contributions

GS and SA-C contributed to conception and design of the study. GS, SA-C, AC-H and KR-C contributed to the generation of data. GS, SA-C, AC-H, CA-S and EA-S contributed to the analysis and interpretation of data. GS, SA-C, AC-H, EA-S, CA-S and JA-G, wrote sections of the manuscript. GS, SA-C, AC-H, EA-S, KR-C, JA-G, CA-S and LM-B contributed to manuscript revision, read, and approved the submitted version.
