# Supplementary figures and images for: Highly purified and functionally stable in vitro expanded allospecific Tr1 cells expressing immunosuppressive graft-homing receptors as new candidates for cell therapy in solid organ transplantation
Source: Front Immunol. 2023 Feb 24;14:1062456. doi: 10.3389/fimmu.2023.1062456 (PMC9998667; doi:10.3389/fimmu.2023.1062456)

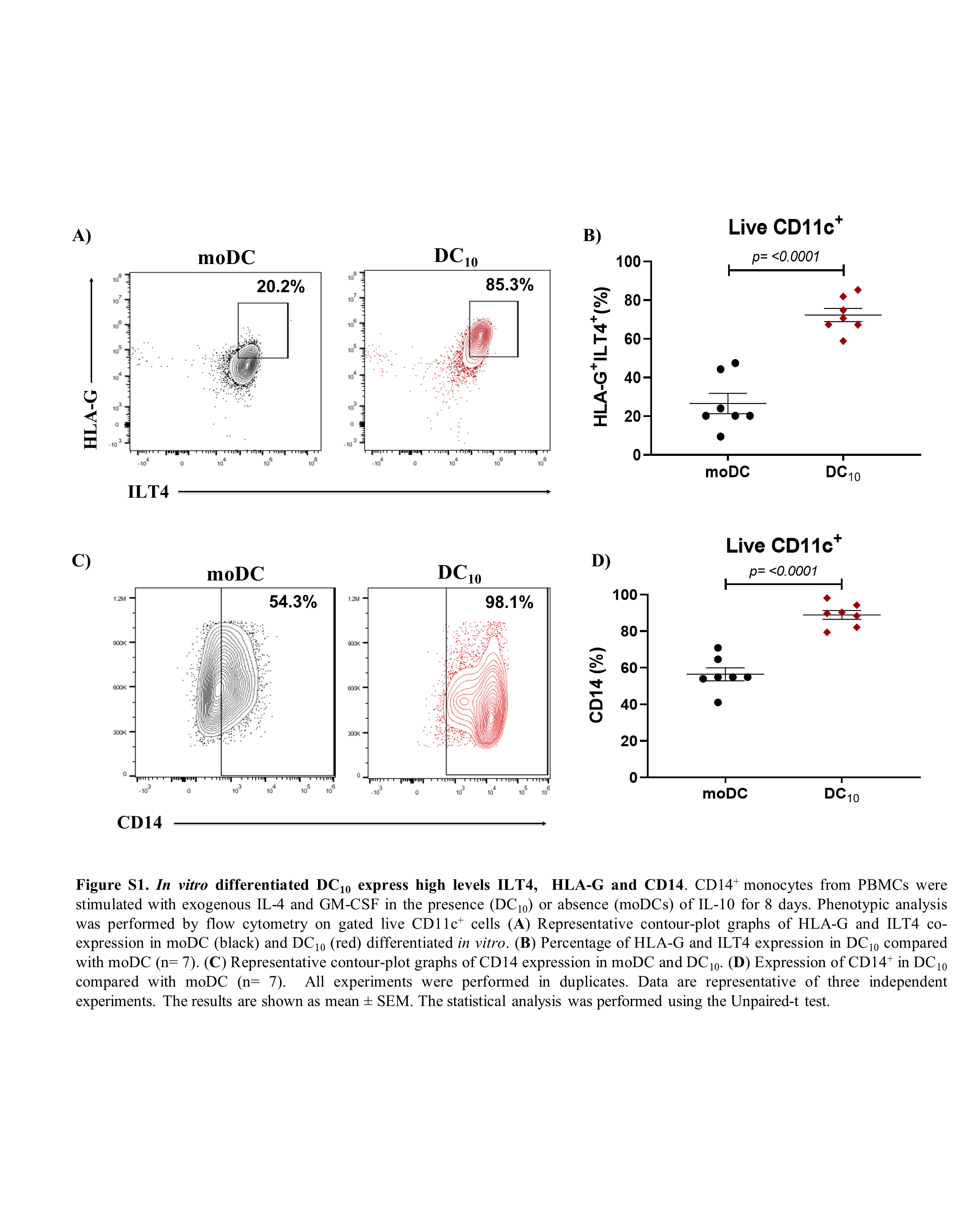

Supplement: Supplementary file 1 [file Image_1.tif]

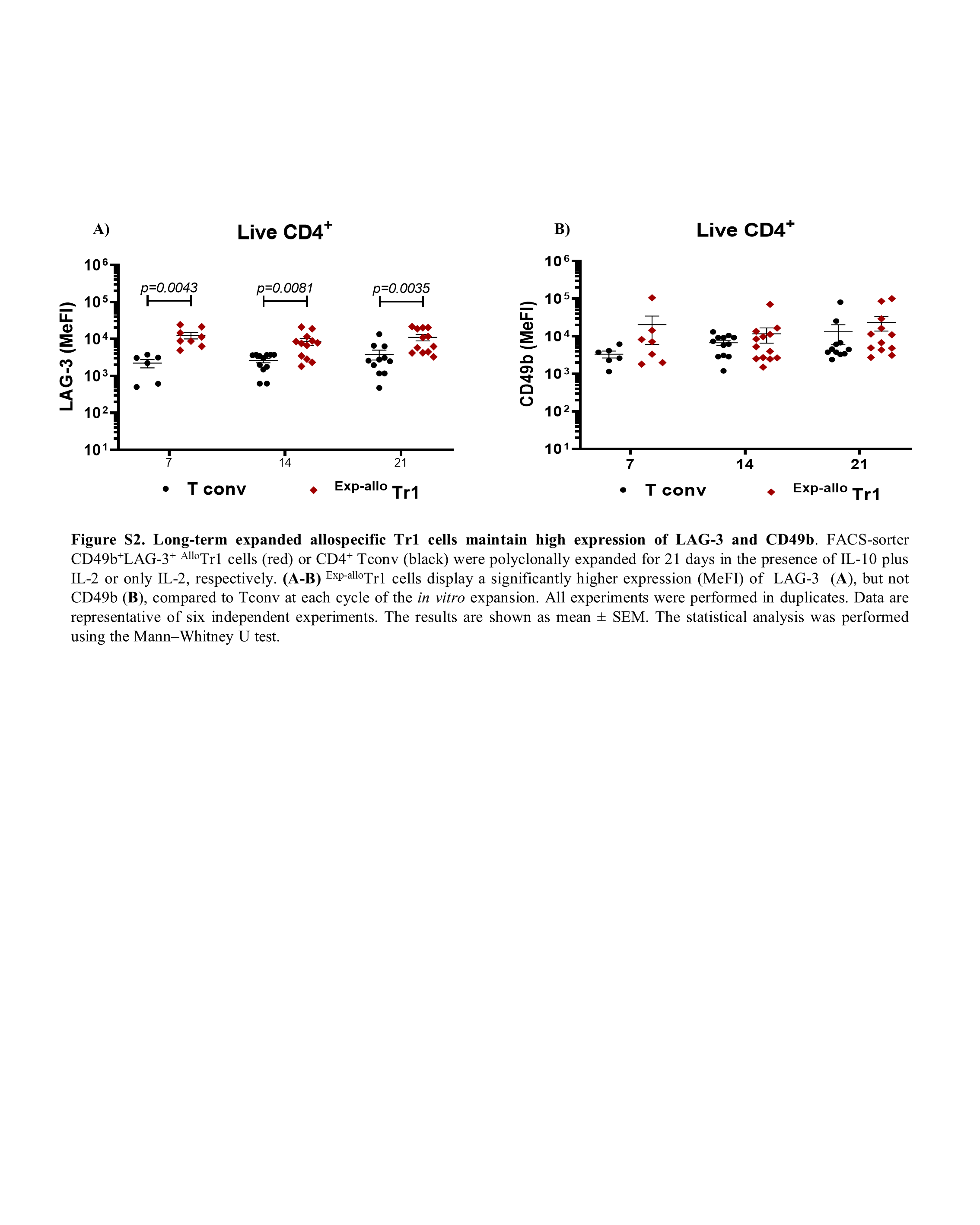

Supplement: Supplementary file 2 [file Image_2.tif]

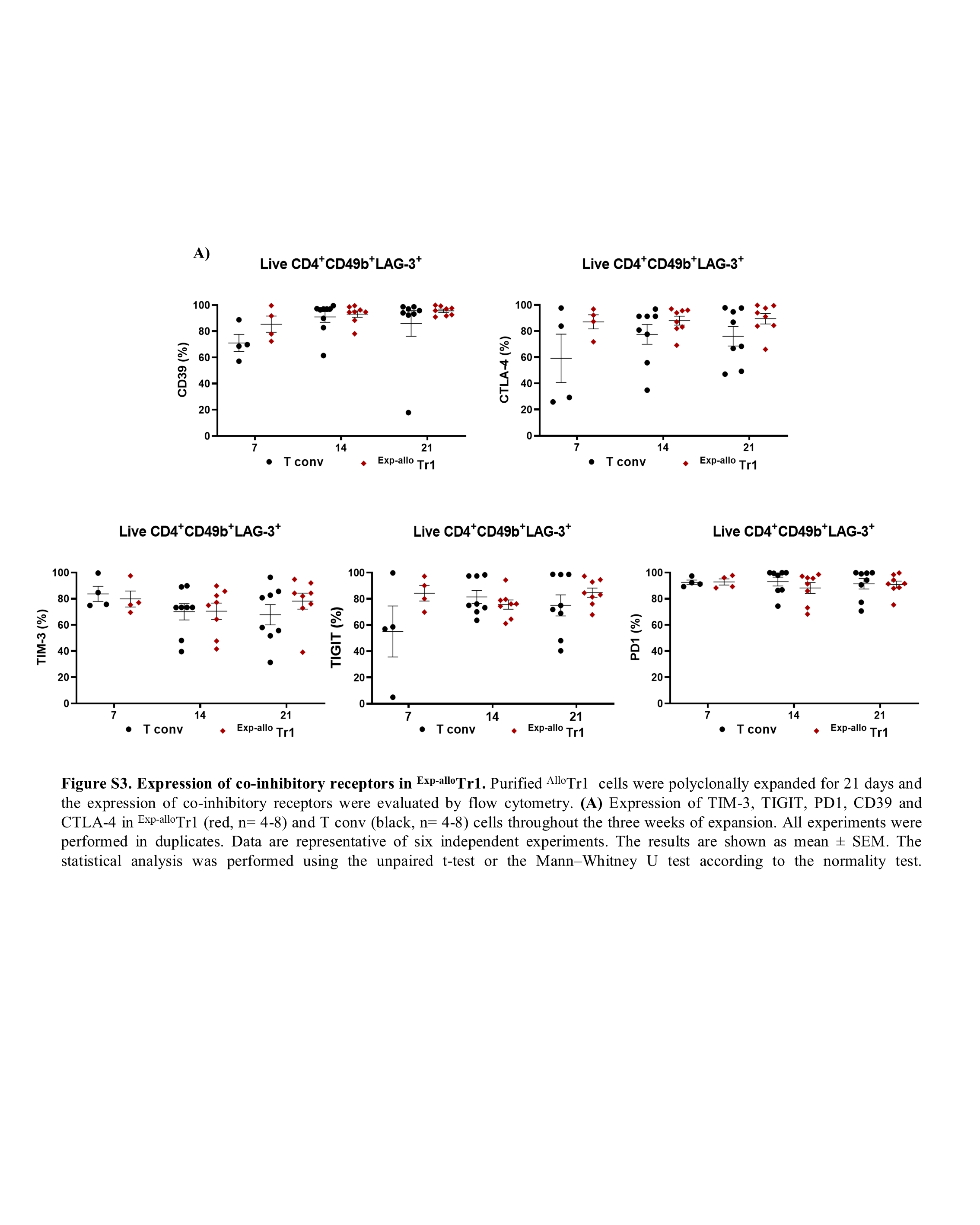

Supplement: Supplementary file 3 [file Image_3.tif]

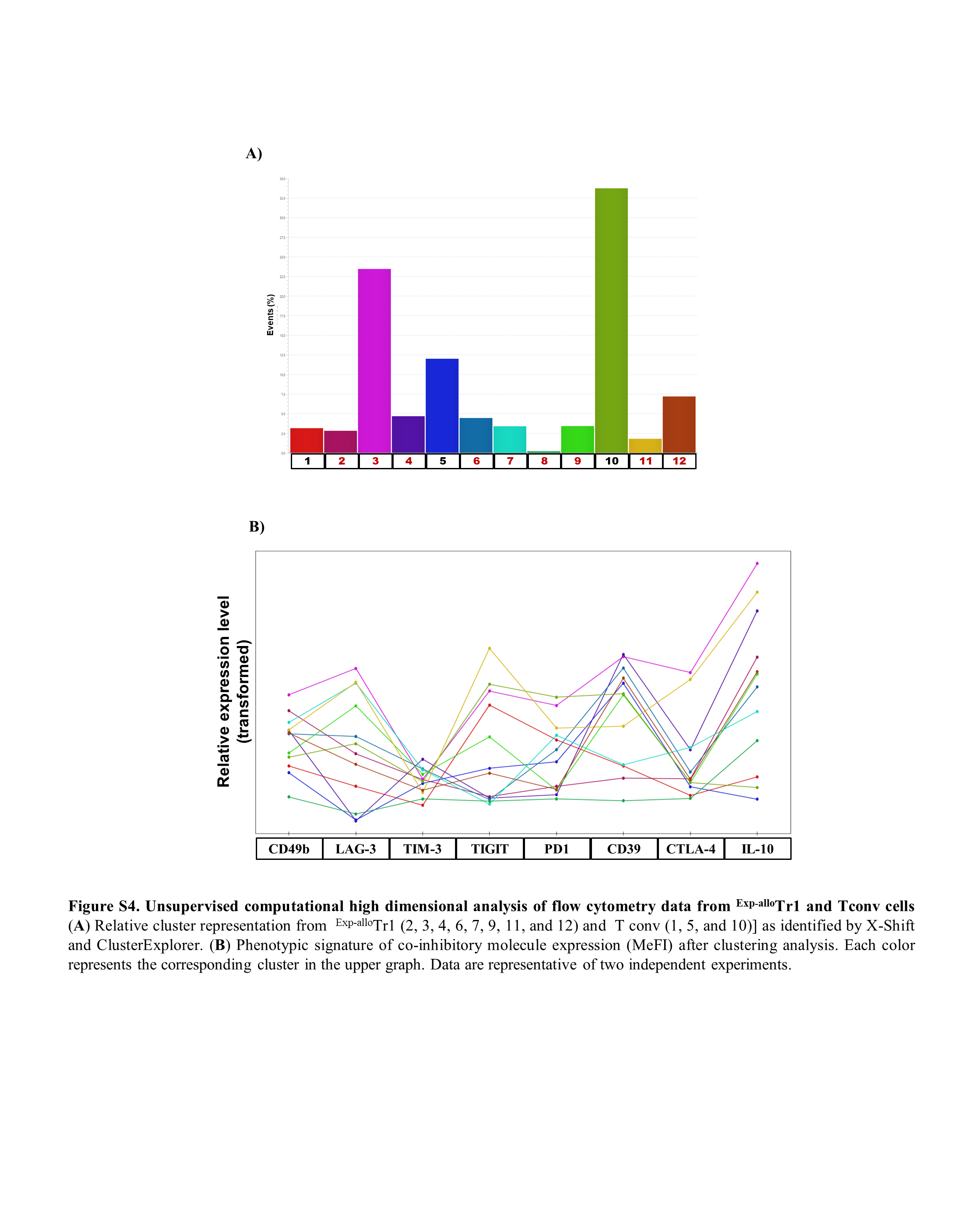

Supplement: Supplementary file 4 [file Image_4.tif]

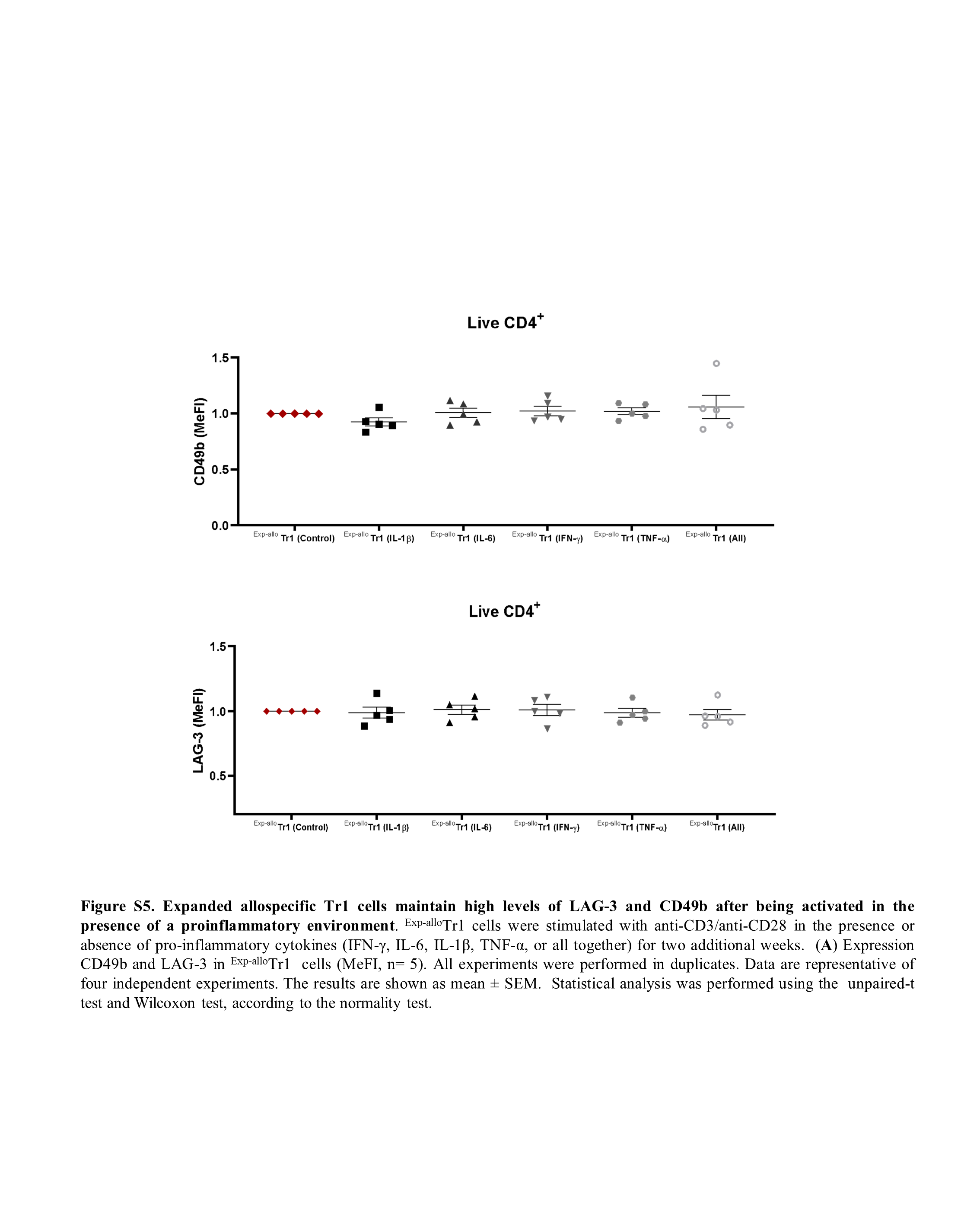

Supplement: Supplementary file 5 [file Image_5.tif]
