# Supplementary material for: Highly purified and functionally stable in vitro expanded allospecific Tr1 cells expressing immunosuppressive graft-homing receptors as new candidates for cell therapy in solid organ transplantation
Source: Front Immunol. 2023 Feb 24;14:1062456. doi: 10.3389/fimmu.2023.1062456 (PMC9998667; doi:10.3389/fimmu.2023.1062456)
Supplement: Supplementary file 6 [file Table_1.pdf]

Reagent	Source (Brand)	Identifier (#Catalog)	Clone
Anti-human CD4 APC-Cy7	Tonbo	25-0049-T100	RPA-T4
Anti-human CD8 PE Cy7	Tonbo	60-0088-T100	RPA-T8
Anti-human CD11c APC	Tonbo	20-0116-T100	3.9
Anti-human CD14 FITC	Tonbo	35-0149-T100	61D3
Anti-human LAG-3 PE Cy7	Invitrogen	2018401	3DS223H
Anti-human CD3 PE-Cy5.5	Invitrogen	35-0036-42	SK7
Anti-human ILT4 PerCP-eFluor 710	Invitrogen	46-5149-41	42D1
Anti-human PD1 Alexa Fluor 488	Biolegend	367408	NAT105
Anti-human CCR5 Alexa Fluor 488	Biolegend	359104	J418F1
Anti-human CCR7 PerCP Cy5.5	Biolegend	353220	G043H7
Anti-human TIM3 PerCP-Cy5.5	Biolegend	345016	F38-2E2
Anti-human PE anti-CD25	Biolegend	302606	BC96
Anti-human TIGIT PE Dazzle 594	Biolegend	372716	A15153G
Anti-human HLA-G PE-Cy7	Biolegend	335912	87G
Anti-human CD49b APC	Biolegend	359310	P1E6-C5
Anti-human CD45RA APC Fire 750	Biolegend	304152	HI100
Anti-human CD39 Brilliant Violet (BV) 711	Biolegend	328228	A1
Anti-human CTLA-4 Brilliant Violet (BV) 421	Biolegend	369606	BNI3
Anti-human CCR4 Brilliant Violet (BV) 421	Biolegend	359414	L291H4
Anti-human CXCR3 Brilliant Violet (BV) 711	Biolegend	353732	G025H7
Zombie NIR™ Fixable Viability Kit	Biolegend	423106	
Zombie Aqua™	Invitrogen	433102	

Table 1. Reagents used for Flow Cytometry assays.
